# Enhancement of Catalytic Activity and Stability of La_0.6_Ca_0.4_Fe_0.7_Ni_0.3_O_2.9_ Perovskite with ppm Concentration of Fe in the Electrolyte for the Oxygen Evolution Reaction

**DOI:** 10.3390/ma14216403

**Published:** 2021-10-26

**Authors:** Sergei V. Porokhin, Victoria A. Nikitina, Artem M. Abakumov

**Affiliations:** Center for Energy Science and Technology, Skolkovo Institute of Science and Technology, Nobel Street 3, 143026 Moscow, Russia; V.Nikitina@skoltech.ru

**Keywords:** oxygen evolution reaction, water splitting, perovskite, lattice oxygen mechanism

## Abstract

The catalytic activity and stability of an iron-nickel based oxygen-deficient perovskite for the oxygen evolution reaction (OER) are drastically improved with the ppm additive of Fe ions to the alkaline electrolyte. The enhancement is attributed to a 1–2 nm restructured Ni_0.5_Fe_0.5_O_x_(OH)_2-x_ (oxy)hydroxide layer, as demonstrated with scanning transmission electron microscopy. La_0.6_Ca_0.4_Fe_0.7_Ni_0.3_O_2.9_ shows almost a four-fold increase in OER activity after Fe addition relative to the as-prepared pristine electrolyte, which demonstrates the low Tafel slope of 44 ± 2.4 mV dec^−1^ and the superior intrinsic activity of 706 ± 71 A g^−1^_oxide_ at 1.61 V vs. RHE.

Efficient water electrolysis is highly desirable for economic branches where pure hydrogen and/or oxygen are used, for instance, in the transport sector with fuel cell electric vehicles, metallurgy (metal processing), and medicine. In the near future, stable constant growth in the consumption of pure hydrogen and oxygen as environmentally friendly sustainable resources is expected [1,2]. To meet the demand for economically viable clean production with renewable energy sources, it is necessary to increase the energy efficiency of water electrolysis by reducing the anodic overvoltage of the oxygen evolution reaction (OER), which is the rate-determining reaction in electrochemical water splitting. 

Layered double hydroxide and perovskite materials have long been regarded as efficient OER electrocatalysts under alkaline conditions [3,4,5,6,7,8]. In particular, Ni-Fe-based oxide catalysts are well-studied and recognized materials for water electrolysis in alkaline media (for OER), which at certain conditions even outperform the electrocatalysts based on noble Ir, Ru elements [9,10]. The mechanism beneath high catalytic performance is related to lattice oxygen evolution reaction (LOER), which is characteristic of complex metal oxides (including perovskites). In light of recent studies [11,12], LOER is now seen as a fundamental process resulting in surface reconstruction towards highly active transition metal (oxy)hydroxides due to shallow perovskite A-site dissolution and B-site cation dissolution/re-deposition [13,14,15,16].

The fact that a small amount of Fe adsorbed from the electrolyte strongly affects OER activity and the stability of catalysts has already been reported for (Ni,Fe,Co)O_x_H_y_ materials [17,18]. Rationalizing this observation is of extreme importance for the further design of efficient OER catalysts, as commercial electrolytes used for alkaline water electrolysis always contain Fe traces (typically around 1 ppm) [19,20], with the Fe content in most cases being poorly controlled under conventional experimental conditions. Recently, it has been shown that the surface of perovskite-type oxides can be deliberately modified with active (oxy)hydroxide layers by the addition of a small amount of Fe^3+^ ions to the electrolyte solution. When (oxy)hydroxide layers are deposited on the surface of oxides such as Ba_0.5_Sr_0.5_Co_0.8_Fe_0.2_O_3−δ_, SrTi_0.1_Fe_0.85_Ni_0.05_O_3−δ_ [16,21,22], and LaNiO_3_ [9,23], OER activity and stability demonstrates synergistic enhancement. This indicates that for perovskite catalysts capable of undergoing LOER-induced surface reconstruction, the Fe species in the electrolyte are an essential component, as these species ensure the dynamically stable active sites in the surface (oxy)hydroxide layers [11,12]. Currently, further studies with the controlled addition of Fe species are needed for correct interpretation of electrochemical behavior of perovskite-based material in terms of activity–stability relationships.

In this work, we report on the enhanced OER catalytic activity of Ca-doped Ni/Fe-mixed perovskite with the additional presence of Fe ions in the electrolyte. This material has been demonstrated to possess promising OER activity due to the beneficial effect of Ca doping, which decreases the formation energy of the oxygen vacancies [24]. In this work, we search for strategies to further increase the activity and long-term stability of this material under the OER conditions. The La_0.6_Ca_0.4_Fe_0.7_Ni_0.3_O_2.9_ (LCFN43) perovskite was prepared with a modified spray pyrolysis approach, as reported previously by our group [24]. Powder X-ray diffraction demonstrates a well-crystallized material with the perovskite-like *R*-3*c* structure with the unit cell parameters *a* = 5.4797(9) Å, *c* = 13.338(4) Å, V = 346.8(1) Å^3^ (See Supplementary Figure S1).

Morphologically, the sample consists of porous hollow spherical particles with diameters ranging between 200 and 1200 (Figure 1) and with a BET specific surface area of 15 m^2^g^−1^. The spheres demonstrate mostly homogeneous cation distribution (Figure 1). The cation atomic ratio measured with energy-dispersive X-ray (EDX) analysis amounts to La:Ca:Fe:Ni = 0.58(1):0.41(1):0.70(1):0.31(1), which is in good agreement with the nominal composition. The oxygen content was determined using iodometric titration. Small particles with apparent Ca excess were also observed with EDX compositional mapping and attributed to the CaO impurity phase. This impurity, however, is washed out when soaking the sample in alkaline electrolyte [24], which allowed us to conduct the electrochemical measurements solely on the perovskite phase.

The OER activity of LCFN43 was evaluated in Ar-saturated 1M NaOH solution. Cyclic voltammograms (CVs) were registered within the potential limits of 0.93–1.66 V vs. RHE. The IR-corrected CVs in Figure 2a,b compare the current densities normalized to the geometric surface area of the rotating disk electrode (RDE). A much higher current density (12.9 mA cm^−2^ at 1.61 V RHE) was observed by adding Fe ions to the electrolyte in 1 ppm concentration, compared to the as-prepared pristine electrolyte (3.0 mA cm^−2^ at 1.61 V RHE). The Tafel slope in the Fe-modified electrolyte is significantly lower (44.0 ± 2.4 mV dec^−1^), indicating faster reaction kinetics compared to 52.0 ± 2.6 mV dec^−1^ in the pristine electrolyte (Figure 2c). 

The LCFN43 perovskite demonstrates a superior OER catalytic activity of 706 ± 71 A g^−1^_oxide_ at 1.61 V vs. RHE, which is comparable to that (assuming 10% uncertainty) for LaNiO_3_ covered with amorphous Ni-Fe (oxy)hydroxide, occurred after post-treatment by FeCl_3_ (755 ± 76 A g^−1^_oxide_) [9] and pristine (Ni-Fe) hydroxides (600 ± 60 A g^−1^_oxide_) [25], and it outperforms other top perovskite catalysts, such as Pr_0.5_Ba_0.3_Ca_0.2_CoO_3-δ_ (85 ± 9 A g^−1^_oxide_) [26] and La_0.4_Sr_0.6_Ni_0.5_Fe_0.5_O_3-δ_ (375 ± 38 A g^−1^_oxide_) [27] (Figure 2d). 

We also measured the initial amount of Fe ions in aqueous 50 wt.% solution of NaOH (Sigma-Aldrich, Saint-Louis, MO, USA) by ICP-AES and observed 1200 µg L^−1^ concentration that corresponds to 63.4 µg L^−1^ (~0.063 ppm) in the as-prepared pristine 1M NaOH electrolyte. Therefore, 1 ppm of Fe is a significant addition that substantially improves the OER activity. Moreover, the potentials of the Ni^4+/3+^ redox peaks were slightly shifted after immersion in 1 ppm Fe_aq_ electrolyte (Figure 2b) [18,29,30], evidencing that Fe incorporates/adsorbs on the surface, increasing the capacity of the cathodic peak, which is related to the formation of a thicker or more redox active Ni-Fe (oxy)hydroxide layer [8,30]. The LCFN43 sample demonstrates an activity improvement factor (ratio of current values in the 1 ppm Fe_aq_ electrolyte and in the pristine electrolyte) equal to 3.9. Such improvement, however, was not achieved immediately, but after 20 h of soaking the LCFN43 electrode in the pristine electrolyte. If the measurements were performed without preliminary soaking or additional cycling, the improvement factor was 2.5 for the very first CV cycles, suggesting that the enhancement depends on pretreatment history, which in turn affects the surface composition [8,25]. For instance, improvement of OER activity in the La_1-x_Sr_x_CoO_3_ (x = 0, 0.3) system steadily rises with cycling in KOH-based electrolyte deliberately containing 0.1 ppm of Fe_aq_ compared to the Fe-free solution, reaching after 1000 cycles the improvement factor of ~4 [12], similar to 3.9 in our case. Additionally, the NiO catalyst has also been tested in similar conditions with added Fe(NO_3_)_3_, demonstrating a 2.3-fold increase in OER activity [31].

To test the effect of Fe addition on the long-term stability of LCFN43 performance, the electrode consisting of perovskite catalyst mixed with 50 wt.% Vulcan carbon XC72R (VC) was polarized galvanostatically in a stepwise mode with the 32.6, 56, 78, 56, and 32.6 A g^−1^_oxide_ current densities for at least 14 h (Figure 3a,b). The lower mass loading compared to the CV tests was used to maximize the homogeneity of catalyst coating, i.e., particle wetting and connectivity in the working environment. The relatively low current densities for the stability test were chosen to avoid high overvoltages, causing VC corrosion and oxygen bubble formation during OER.

The potential slope slightly increases during the constant current test in a stepwise mode at short experimental times (up to 3900 s, Figure 3b). Further, the slope continues increasing and finally stabilizes at ~15,500 s, which suggests the shallow A-cation leaching and restructuring of the surface at the initial stage [24] and then the formation of dynamic equilibrium between Fe dissolution from perovskite during OER and redeposition promoted by Fe_aq_ in the electrolyte. This dynamic stability of active sites (Fe exchange) prevents deep structural changes of the perovskite particles [11,12]. After 21,000 s, the overpotential starts decreasing continuously, and this suggests an increase in the number of active surface centers that enhance the OER activity. A completely different behavior with a sharp increase in overpotential during galvanostatic polarization was observed for LCFN43 without the addition of Fe to the electrolyte (Figure S2).

In order to obtain deeper insight into the changes of the perovskite surface, the LCFN43 sample was collected directly from the electrode after the constant current test. The electrode was rinsed with deionized water, then with isopropanol, and then the thin catalyst film was scraped off with a micropipette and further transferred onto the TEM grid. 

On the very surface of the perovskite particles after the constant current test, a thin restructured layer is observed (Figure 4a). The layer is 1–2 nm thick; it is not entirely amorphous and contains ordered nanoparticles. One of the nanoparticles (outlined in Figure 4a and enlarged in Figure 4b) demonstrates a hexagonal arrangement of cationic columns with variable intercolumn distance with an average value of 2.9(3) Å. This corresponds well to the 001 projection of the *P*-3*m*1 crystal structure of Ni(OH)_2_ layered hydroxide [32]. In fact, the measured projected distance between the cationic columns is in between those in Ni(OH)_2_ (3.13 Å) and in NiOOH (2.81 Å), which reflects that the actual structure is in between of hydroxide and oxyhydroxide. EDX-STEM compositional maps and intensity profiles demonstrate that the surface layer contains both Ni and Fe (Figure 4c,d) with the Ni:Fe ratio of 54(3):46(3). Thus, the restructured surface layer can be classified as a mixed Ni_0.5_Fe_0.5_O_x_(OH)_2-x_ (oxy)hydroxide with variable O/OH ratio.

The CV and galvanostatic tests, along with the TEM data, allow us to conclude that the (oxy)hydroxide layer formed on the host (perovskite) surface under the OER conditions is responsible for the enhanced OER activity. The presence of Fe in the electrolyte stabilizes the dynamic active OER sites and prevents deep structural changes of the perovskite particles. In summary, the addition of 1 ppm of Fe into alkaline electrolyte creates a stable dynamic (oxy)hydroxide interface with the host perovskite catalyst, thus increasing both the catalytic activity and the stability of the catalyst in oxygen evolution reaction.

## Figures and Tables

**Figure 1:**
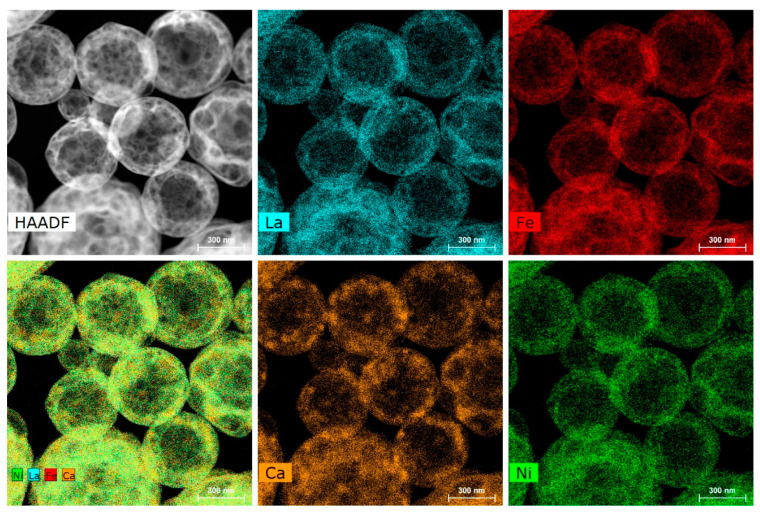
HAADF-STEM image of spherical hollow particles in the LCFN43 sample along with the La, Ca, Fe, Ni STEM-EDX maps and a mixed color-coded compositional map.

**Figure 2:**
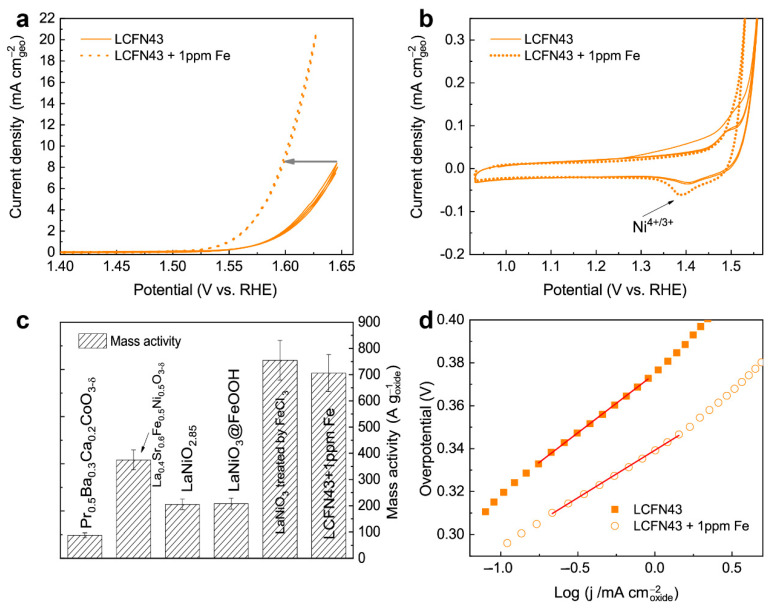
(**a**) CVs of the LСFN43 perovskite in the as-prepared pristine electrolyte and with the addition of 1 ppm Fe_aq_, normalized to the geometric area of the electrode. (**b**) Enlarged regions of the CVs. (**c**) Mass activities at 1.61 V vs. RHE of LCFN43 + Fe_aq_ and comparison with other OER catalysts [9,23,26,27,28]. (**d**) Tafel plots for LСFN43 in the as-prepared pristine electrolyte and with the addition of 1 ppm Fe_aq_. Measurement conditions: Ar-saturated 1M NaOH solution at 10 mV s^−1^ and 1600 rpm, total mass loading, 35.7 µg cm^−2^, 50 wt.% Vulcan carbon XC72R.

**Figure 3:**
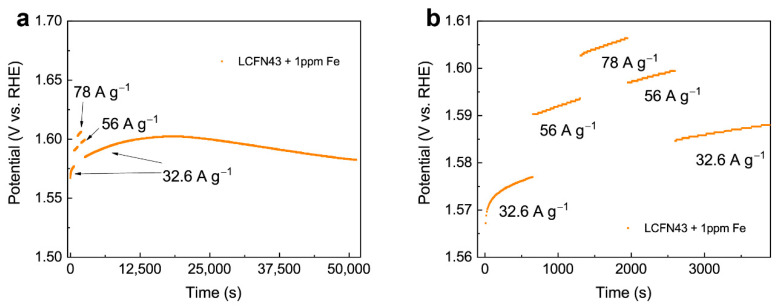
(**a**) Constant current test on LCFN43 in 1 ppm Fe_aq_ electrolyte at stepwise changes of the current density from 32.6 to 56, 78, and back to 56 and 32.6 A g^−1^_oxide_. (**b**) Enlarged regions of the first 3900 s of the test. Measurement conditions: Ar-saturated 1M NaOH solution, 1600 rpm, mass loading 18 µg cm^−2^, 50 wt.% VC.

**Figure 4:**
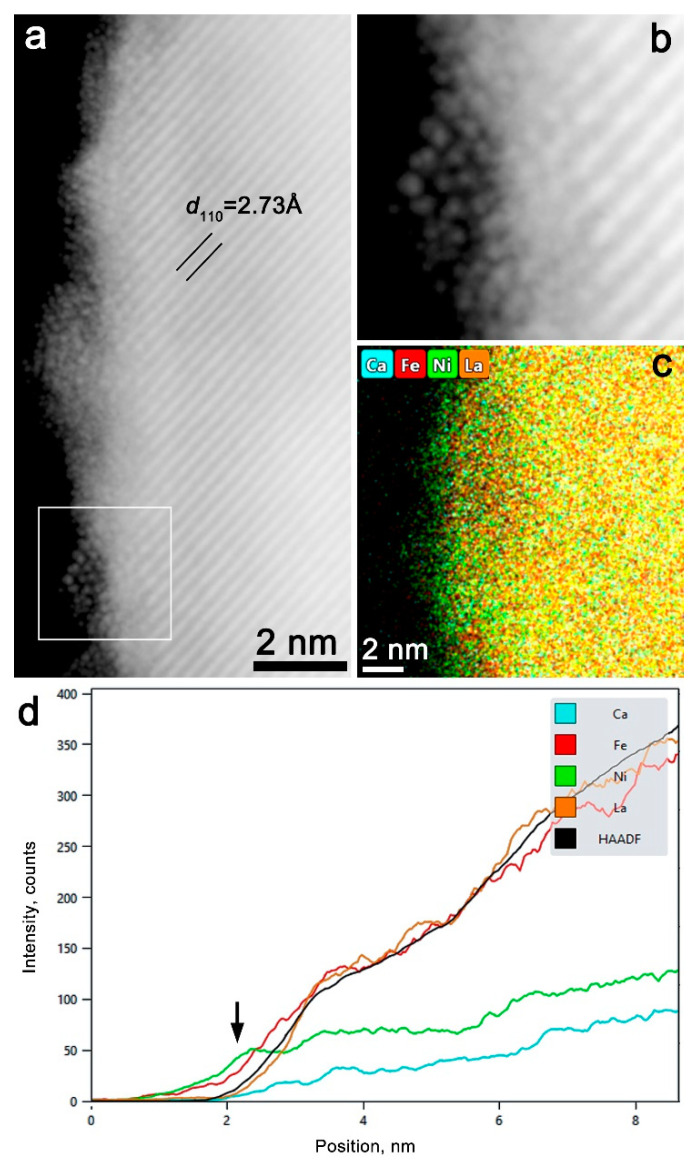
(**a**) HAADF-STEM image of the near-surface area in the LCFN43 sample after 14 h constant current test. The LCFN43 particle is visible by traces of the {110} crystal planes of the perovskite sub-cell with d ≈ 2.73 Å. A restructured surface layer with a thickness of 1–2 nm is visible. (**b**) Enlargement of the 001-oriented (Ni,Fe) (oxy)hydroxide nanoparticle in the restructured layer. (**c**,**d**) EDX-STEM compositional map and intensity profiles demonstrate that the surface layer (marked with the arrow) is Ni, Fe—enriched and La, Ca—depleted.

## Data Availability

Data available on request.

## Supplementary Materials

The following are available online at https://www.mdpi.com/article/10.3390/ma14216403/s1, Figure S1. PXRD profiles after Rietveld refinement of the pristine La_0.6_Ca_0.4_Fe_0.7_Ni_0.3_O_2.9_ perovskite. The ticks indicate the Bragg reflection positions for the main perovskite phase (bottom row) and the CaO admixture (4.4(5) wt.%, top row). Figure S2. Constant current tests on LCFN43 in the electrolyte containing ppm amounts of Fe_aq_ (dark line) and in the as-prepared electrolyte (bright line). Experimental conditions: Ar-saturated 1M NaOH solution, 1600 rpm, mass loading 35.7 µg cm^−2^, 50 wt.% VC. Materials and method: Synthesis, Powder X-ray diffraction, Transmission electron microscopy, Surface area analysis, Electrochemical measurements, Inductively coupled plasma atomic emission spectroscopy.
