# Supplementary material for: Enhancement of Catalytic Activity and Stability of La0.6Ca0.4Fe0.7Ni0.3O2.9 Perovskite with ppm Concentration of Fe in the Electrolyte for the Oxygen Evolution Reaction
Source: Materials (Basel). 2021 Oct 26;14(21):6403. doi: 10.3390/ma14216403 (PMC8585416; doi:10.3390/ma14216403)
Supplement: Supplementary file 1 [file materials-14-06403-s001.zip › materials-1422103-supplementary.pdf]

Supplementary Materials

Enhancement of Catalytic Activity and Stability of $La_{0.6}Ca_{0.4}Fe_{0.7}Ni_{0.3}O_{2.9}$ Perovskite with ppm Concentration of Fe in the Electrolyte for the Oxygen Evolution Reaction

Sergei V. Porokhin *, Victoria A. Nikitina and Artem M. Abakumov *

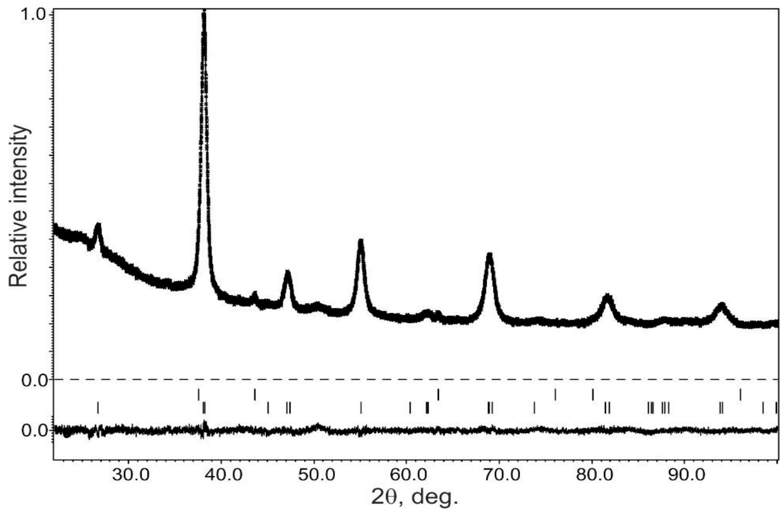

Figure S1. PXRD profiles after Rietveld refinement of the pristine $La_{0.6}Ca_{0.4}Fe_{0.7}Ni_{0.3}O_{2.9}$ perovskite. The ticks indicate the Bragg reflection positions for the main perovskite phase (bottom row) and the CaO admixture (4.4(5) wt.%, top row).

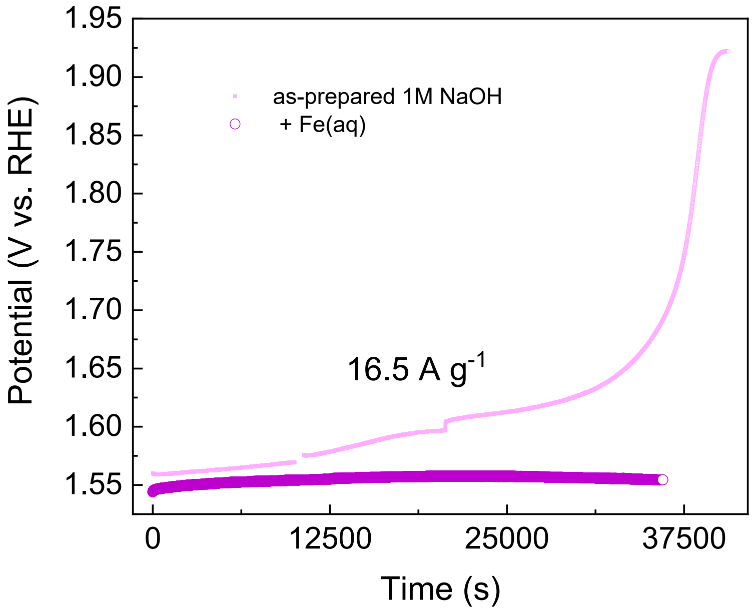

Figure S2. Constant current tests on LCFN43 in the electrolyte containing ppm amounts of Fe_{aq} (dark line) and in the as-prepared electrolyte (bright line). Experimental conditions: Ar-saturated 1M NaOH solution, 1600 rpm, mass loading 35.7 μg cm^{-2}, 50 wt.% VC.

Citation: Porokhin, S.V.; Nikitina, V.A.; Abakumov, A.M. Enhancement of Catalytic Activity and Stability of $La_{0.6}Ca_{0.4}Fe_{0.7}Ni_{0.3}O_{2.9}$ Perovskite with ppm Concentration of Fe in the Electrolyte for the Oxygen Evolution Reaction. *Materials* **2021**, *14*, 6403. https://doi.org/10.3390/ma14216403

Academic Editor: Alberto Vertova

Received: 29 September 2021
Accepted: 22 October 2021
Published: 26 October 2021

Publisher's Note: MDPI stays neutral with regard to jurisdictional claims in published maps and institutional affiliations.

Copyright: © 2021 by the authors. Submitted for possible open access publication under the terms and conditions of the Creative Commons Attribution (CC BY) license (http://creativecommons.org/licenses/by/4.0/).

In the electrolyte without the intentional addition of Fe ions the potential steadily rises reaching saturation at 40,000 s. This behavior can be associated with the the dissolution of Fe from perovskite during OER, which does not redeposit from the electrolyte due to the very low concentrations. Under these conditions, the restructured surface layers are unable to provide sufficient amount of active sites [1,2], which results in an increase of the overpotential and VC corrosion provoked by high overpotentials [3].

Materials and Methods

Synthesis

The LC4FN3 perovskite was synthesized using the modified ultrasonic spray pyrolysis (USP) method. The nitrates $La(NO_3)_3 \cdot 6H_2O$ (Neva Reactive, ≥99.0%), $Ca(NO_3)_2 \cdot 4H_2O$ (Neva Reactive, ≥98.5%), $Fe(NO_3)_3 \cdot 9H_2O$ (Alfa Aesar, Haverhill, MA, USA, >98.0%) and $Ni(NO_3)_2 \cdot 6H_2O$ (Sigma Aldrich, Saint Louis, MO, USA, ≥97.0%) were dissolved in deionized water (MilliQ, Merck, Kenilworth, NJ, USA, 18.2 MΩ) in the appropriate molar stoichiometry to achieve the total concentration of 10 wt.%. Sorbitol $C_6H_{14}O_6$ (Sigma Aldrich, ≥99.0%) was added at 0.78 wt.% from total concentration of aqueous solution. The solution was ultrasonically sprayed using a household ultrasonic humidifier at ν = 2.64 MHz and transferred with an air flow ($V_{air\ flow}$ = 172 mL s^{-1}) together with ozon O_3 flow produced with the rate of 400 mg h^{-1} into a tubular furnace pre-heated to 850 °C with the hot zone length of 360 mm. The resulting powder was collected with a Schott funnel with a glassy G4 filter. Then the powder was additionally annealed at 600 °C for 5 h in air and furnace-cooled.

Powder X-ray Diffraction

Phase purity and the lattice parameters were determined by powder X-ray diffraction (PXRD) using a Huber G670 Guinier camera, Huber, Edison, NJ, USA (transmission geometry, curved primary Ge (111) monochromator, Co $K_{\alpha 1}$ radiation, λ = 1.78896 Å, image plate detector).

Transmission Electron Microscopy

The samples for transmission electron microscopy (TEM) were prepared by grinding the powders with an agate mortar and pestle in ethyl alcohol and depositing drops of suspension onto a carbon film supported by a copper grid. High angle annular dark field scanning transmission electron microscopy (HAADF-STEM) images and energy-dispersive X-ray STEM-EDX compositional maps were acquired on a probe aberration-corrected FEI Titan Themis Z transmission electron microscope, Thermo Fisher, Waltham, MA, USA at 200 kV equipped with a Super-X system for EDX analysis.

Surface Area Analysis

Nitrogen adsorption analysis was performed with a Quantachrome Instruments NOVA 2000, Quantachrome Instruments, Boynton Beach, FL, USA high-speed surface area analyzer at 77 K. Prior to the measurements, the samples were degassed in dynamic vacuum for at least 12 h at 120 °C. The specific surface area was calculated using the Brunauer-Emmett-Teller (BET) method from the nitrogen adsorption data in the relative pressure range (P/P_0) from 0.05 to 0.30. Only the adsorption branch of the isotherm was used for the analysis.

Catalyst Ink Deposition

Oxide powders were deposited onto glassy carbon (GC) disk electrodes as thin films. GC electrodes were polished with 0.05 μm Al_2O_3 slurry on a polishing cloth, rinsed with distilled water several times, and then dried under an air stream. The appropriate amounts of perovskite powder and Vulcan carbon XC72R (VC) were added to a water/isopropanol solution (4:1 by volume), which contained 0.06 wt.% of Na-substituted Nafion (Sigma Aldrich, Saint-Louis, MO, USA, 117 solution) to reach ca. 1 $g_{Nafion}\ g^{-1}_{catalyst}$. The resulting mixture was sonicated for 30 min to obtain a homogeneous ink. To deposit uniform catalyst layers, three portions (~3.9 μL each) of the ink were subsequently drop-cast onto the GC disk electrode using a micropipette with intermediate drying under a weak Ar gas stream. The total catalyst (oxide plus Vulcan carbon) loadings were 35.7 μg cm^{-2}.

Electrochemical Measurements

Electrochemical measurements were performed using a Metrohm Autolab PGSTAT302N bipotentiostat (Metrohm Autolab, Utrecht, Netherlands) equipped with a linear scan generator module. The working electrode was a Metrohm rotating disk electrode (RDE) GC disk electrode with a 3 mm diameter. All the electrochemical measurements were performed in a three-electrode Teflon cell filled with 1 M NaOH at 25 °C with temperature control by the water-cooled thermostatic bath. The reference electrode was Hg/HgO (1 M NaOH, Pine Research Instruments, Durham, NC, USA), platinum wire was used as counter electrodes. All potentials reported here were recalculated to the reversible hydrogen electrode (RHE) scale via the equation: E(RHE) = E(Hg/HgO in 1M NaOH) + 0.930 V. 1 M NaOH solution was prepared from 50 wt.% NaOH aqueous solution (Sigma-Aldrich).

In order to evaluate the catalyst's performance in the electrolyte after the addition of Fe_{aq}, aqueous solution of $Fe(NO_3)_3*9H_2O$ was added to the 50 wt.% NaOH solution (Sigma-Aldrich) to reach Fe_{aq} concentration of 1 ppm in the 1 M NaOH electrolyte + Fe.

For the OER kinetic measurements, linear sweep cyclic voltammograms (CVs) at 10 mV s^{-1} were recorded in Ar-saturated 1M NaOH solution at a 1600 rpm rotation rate. The uncompensated ohmic resistance values (10–20 Ω) were determined from the high-frequency intercept of electrochemical impedance hodographs of RDE electrode recorded at OER potentials in the 0.1–100 kHz frequency range (5 mV amplitude). All the reported RDE voltammograms were IR-corrected.

Inductively Coupled Plasma Atomic Emission Spectroscopy

For the inductively coupled plasma atomic emission spectroscopy (ICP-AES) Perkin Elmer Optima 5300 experiment, the aliquots of the solution were taken from 50 wt.% Sigma-Aldrich aqueous solution to determine the concentration of the Fe traces.

References

1. Klaus, S.; Cai, Y.; Louie, M.W.; Trotochaud, L.; Bell, A.T. Effects of Fe Electrolyte Impurities on Ni(OH) 2 /NiOOH Structure and Oxygen Evolution Activity. *J. Phys. Chem. C* **2015**, *119*, 7243–7254, doi:10.1021/acs.jpcc.5b00105.
2. Chung, D.Y.; Lopes, P.P.; Farinazzo Bergamo Dias Martins, P.; He, H.; Kawaguchi, T.; Zapol, P.; You, H.; Tripkovic, D.; Strmcnik, D.; Zhu, Y.; et al. Dynamic stability of active sites in hydr(oxy)oxides for the oxygen evolution reaction. *Nat. Energy* **2020**, *5*, 222–230, doi:10.1038/s41560-020-0576-y.
3. Filimonenkov, I.S.; Bouillet, C.; Kéranguéven, G.; Simonov, P.A.; Tsirlina, G.A.; Savinova, E.R. Carbon materials as additives to the OER catalysts: RRDE study of carbon corrosion at high anodic potentials. *Electrochim. Acta* **2019**, *321*, doi:10.1016/j.electacta.2019.134657.
